# Silver linings of the coronavirus disease 2019 (COVID-19) pandemic from an infection prevention and control perspective

**DOI:** 10.1017/ice.2020.1310

**Published:** 2020-11-04

**Authors:** Kara K. Tsang, Dominik Mertz, Cindy O’Neill, Sarah Khan

**Affiliations:** 1 Department of Biochemistry and Biomedical Sciences, McMaster University, Hamilton, Ontario, Canada; 2 Department of Medicine, Infection Prevention and Control, McMaster University, Hamilton Health Sciences, Hamilton, Ontario, Canada; 3 Hamilton Health Sciences, Hamilton, Ontario, Canada; 4 Department of Pediatrics, Infection Prevention and Control, McMaster University, Hamilton Health Sciences, Hamilton, Ontario, Canada

*To the Editor—*As a response to the evolving information about the coronavirus disease 2019 (COVID-19) pandemic, health agencies and organizations have been updating guidelines for infection prevention and control. McMullen et al^1^ made some predictions about increasing and decreasing hospital-acquired infection rates due to the crisis care for COVID-19. Despite the numerous infection control challenges posed by severe acute respiratory coronavirus virus 2 (SARS-CoV-2) in acute-care facilities, in some ways, we have been able to identify some “silver linings” or opportunities for improved infection control practices amidst the pandemic.

## Quality assurance of personal protective equipment (PPE) practices

Previous to the COVID-19 pandemic, our hospital followed the Public Health Ontario Provincial Infectious Diseases Advisory Committee’s recommendations for different masks and uses, which did not delineate which ASTM mask levels were appropriate. The American Society for Testing and Materials International is a standards organization that develops and publishes consensus technical standards used in the production and testing of personal protective equipment.^2^ Due to the global shortage of PPE supplies, we were able to vet all of our PPE stocks to assess their quality. As part of this audit, we uncovered facemasks in widespread circulation that were not ASTM rated for fluid resistance; these were subsequently removed from our clinical areas. Furthermore, as part of this detailed review of our PPE supplies, we developed standardization in ASTM mask levels appropriate for clinical areas based on the likelihood of facial fluid splash, and for most areas, an ASTM level 1 mask was considered acceptable. This exercise would never have been conducted if the pandemic had not resulted in the development of a PPE task force to review our current inventory and our standard stocking practices.

Concerning the empowerment of staff in their PPE and choices, as part of the pandemic PPE training, all staff were instructed on the process of a point-of-care risk assessment. Despite this being a longstanding aspect of orientation for all staff, only in the pandemic has this become regular verbiage among the staff, indicating effective uptake of basic infection control principles that had previously remained aloof concepts. Similarly, a longstanding principle is that any aerosol-generating medical procedure (AGMP) requires a mask plus eye protection routinely, and a N95 respirator is also required if COVID-19 is suspected.^3^ Furthermore, eye protection has not been generally used within operating rooms, intensive care units (ICU), and emergency departments (EDs) across the province for multiple AGMPs, including intubations (personal communication with Chris Simpson, Queen’s University). With the pandemic, however, uptake of eye protection for such scenarios has significantly improved. Similarly, environmental controls that should have been in place, including plexiglass or a physical barrier^4^ to protect staff from droplet transmission from any virus exposure when encountering unscreened patient populations were implemented. Similarly, screening for febrile respiratory tract infection is performed more systematically and is now the determinant of implementing additional precautions. In contrast, screening may not have previously been as methodical, and precautions often were initiated only if a pathogen was identified from a nasopharyngeal swab.

## Increased hand hygiene compliance rates

In addition to the new systematic guidelines for PPE, we observed improved hand hygiene compliance rates during the COVID-19 pandemic. Between January and April 2020, hand hygiene compliance for Moments 1 and 4 have improved compared to September 2018–December 2019 (Table 1). We suspect that the COVID-19 pandemic has introduced additional concern among healthcare workers, who have become more likely to comply with proper hand hygiene practices for their own safety and that of their patients.

**Table 1.** Hand Hygiene Compliance for Moments 1 Through 4 Between January and April 2020

Type of Indication	September 2018–2019 Compliance, No. (%)	January–April 2020 Compliance, No. (%)	χ^2^	*P* Value
**Moment 1.** Before initial patient-to-patient environmental contact	3,606/4,398 (82.0)	677/793 (85.4)	5.32	.02
**Moment 2.** Before aseptic procedure	284/557 (51)	30/107 (28.0)	35.64	<.00001
**Moment 3.** After body fluid exposure risk	542/816 (66.4)	88/145 (60.7)	1.79	.18
**Moment 4.** After patient-to-patient environmental contact	7,432/8,673 (85.4)	1,400/1,588 (88.2)	6.83	.009

## Decrease in rates of hospital-acquired infections

In terms of hospital-acquired infections, methicillin-resistant *Staphylococcus aureus* (MRSA), vancomycin-resistant *Enterococci* (VRE), extended-spectrum ß-lactamase (ESBL), and carbapenemase-producing *Enterobacteriaceae* (CRE) infection rates per 1,000 patient days in 2020 (January–April) were lower compared to the previous year. Specifically, at one of our hospital sites, the rates per 1,000 patient days for VRE and ESBL bacteremia infections have also decreased compared to the previous year. Thus, the decrease in infection rates could also be attributed to lower bed occupancy between January and April 2020. Lastly, as part of continuous quality improvement huddles in wards, the environmental aids and cleaning staff are becoming increasingly part of infection prevention conversations. Data on cleaning auditing results are being presented more often, and staff have a heightened awareness of the potential for fomite spread, despite it being a small component of COVID transmissions. Ontario health guidelines around optimization of physical distancing, as it pertains to SARS-CoV-2 control, have recommended against the use of 4-bed ward rooms, which has been a source for patient-to-patient transmissions and outbreaks of other healthcare-associated infections due to crowding and to limited ability to clean between patient equipment and belongings. This change may contribute to reductions in the spread of antimicrobial-resistant organisms and to other healthcare-associated infection outcomes.

In summary, despite the global challenges posed by the COVID-19 pandemic for infection preventionists, some valuable short-term gains have been made during this time. However, as hospitals now being to ‘reopen’ to more typical volumes, we may lose ground in areas where we have been able to improve practices. For now, we should celebrate these small wins, and we should aim to continue this culture shift in the minds of frontline workers, prioritizing infection control, PPE practices, and environmental resources that have been put in place. It will be important to be mindful of the potential for ‘mask fatigue’ and for efforts to relax as these public health efforts ‘flatten the curve.’
